# Rates of newly diagnosed breast cancer at commission on cancer facilities during the early phase of the COVID‐19 pandemic

**DOI:** 10.1002/cam4.6874

**Published:** 2023-12-22

**Authors:** Marie Fefferman, Kristine Kuchta, Chi‐Hsiung Wang, Kyra Nicholson, Katherine Kopkash, Catherine Pesce, Elizabeth Poli, Thomas W. Smith, Katharine Yao

**Affiliations:** ^1^ Department of Surgery NorthShore University Health System, Evanston Hospital Evanston Illinois USA; ^2^ Department of Surgery University of Chicago, Pritzker School of Medicine Chicago Illinois USA

**Keywords:** breast neoplasms, COVID‐19, National Cancer Database, pandemic, SARS‐CoV‐2

## Abstract

**Introduction:**

The objective of this study was to examine the impact of the early part of the COVID‐19 pandemic on the number of newly diagnosed breast cancer cases at Commission on Cancer (CoC)‐accredited facilities relative to the United States (U.S.) population.

**Methods:**

We examined the incidence of breast cancer cases at CoC sites using the U.S. Census population as the denominator. Breast cancer incidence was stratified by patient age, race and ethnicity, and geographic location.

**Results:**

A total of 1,499,806 patients with breast cancer were included. For females, breast cancer cases per 100,000 individuals went from 188 in 2015 to 203 in 2019 and then dropped to 176 in 2020 with a 15.7% decrease from 2019 to 2020. Breast cancer cases per 100,000 males went from 1.7 in 2015 to 1.8 in 2019 and then declined to 1.5 in 2020 with a 21.8% decrease from 2019 to 2020. For both females and males, cases per 100,000 individuals decreased from 2019 to 2020 for almost all age groups. For females, rates dropped from 2019 to 2020 for all races and ethnicities and geographic locations. The largest percent change was seen among Hispanic patients (−18.4%) and patients in the Middle Atlantic division (−18.6%). The stage distribution (0–IV) for female and male patients remained stable from 2018 to 2020.

**Conclusion:**

The first year of the COVID‐19 pandemic was associated with a decreased number of newly diagnosed breast cancer cases at Commission on Cancer sites.

## Graphical abstract

This study shows that newly diagnosed breast cancer cases dropped substantially during the first year of the pandemic. The biggest drop was shown for Hispanic patients and patients living in the Middle Atlantic division of the United States. The COVID‐19 pandemic had a large impact on patients with breast cancer and future studies are needed to understand the pandemic's full effect.

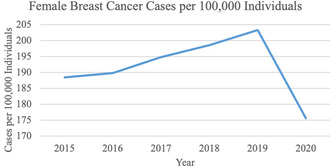

## 1 INTRODUCTION

In January 2020, the United States (U.S.) Secretary of Health and Human Services declared a public health emergency in response to the COVID‐19 pandemic.^1^ Numerous health and government organizations recommended reducing patient contact with the health care system and delaying elective surgeries.^2^, ^3^ The COVID‐19 pandemic changed patient behavior and introduced a period of time during which basic health care was delayed. At the apex of the pandemic in early 2020, health care was limited and, in some areas across the country, unavailable.

Studies have shown that the pandemic had an adverse impact on cancer care.^4^, ^5^, ^6^ Screening for cancer decreased dramatically during the pandemic with an estimated screening deficit of 9.4 million tests for breast, colorectal, and prostate cancer.^7^ This drop in screening was accompanied by a decrease in the number of new cancer cases.^8^, ^9^, ^10^ International studies have also demonstrated this trend with a decrease in the number of newly diagnosed cancer cases of up to 40% during the pandemic.^11^, ^12^, ^13^, ^14^, ^15^, ^16^, ^17^

Despite a well‐documented decrease in many types of cancer diagnoses across the U.S., less is known about breast cancer  during the pandemic. The Commission on Cancer (CoC) was formed in 1922 as a consortium of professional organizations dedicated to improving the care of cancer patients. All CoC‐accredited facilities submit data on their cancer patients to the National Cancer Data Base (NCDB). Accounting for approximately 1500 CoC‐accredited sites across the country, the NCDB represents an ideal source to examine the impact of the COVID‐19 pandemic on breast cancer incidence. The objective of this study was to examine the impact of the first year of the COVID‐19 pandemic on breast cancer incidence across CoC‐accredited centers using the U.S. population as a denominator. This study also examined if patients of a certain age group, race or ethnicity, or geographic region were differentially impacted by the pandemic.

## 2 METHODS

### 2.1 Study design

This was a retrospective cohort study of the incidence of breast cancer among patients treated at CoC centers.

### 2.2 Setting

Females and males ≥18 years old who were diagnosed with invasive and noninvasive breast cancer from 2015 to 2020 were identified in the NCDB. U.S. Census data was used to determine the number of females and males in the general population over the study period. The NCDB does not contain identifiable patient, facility, or provider information; therefore, Institutional Review Board approval was not required for this study given the use of a de‐identified dataset.

### 2.3 Participants

Of the 3,690,015 individuals in the NCDB user file, 1,499,806 patients with stage 0–IV breast cancer were included in the study. There were 1328 unique CoC sites. Cancer cases were selected from the NCDB based on its volume and scope. The U.S. Census population dataset was used as a control to adjust for geographical population change over the study period.

### 2.4 Variables

The exposure was the presence of the COVID‐19 pandemic in 2020. Primary outcome measures were the number of newly diagnosed breast cancer cases from 2015 to 2020 and newly diagnosed breast cancer cases per 100,000 individuals based on U.S. Census data during the same time period. Patients in the NCDB were analyzed based on available demographic and diagnostic information, including sex, age, race and ethnicity, year of cancer diagnosis, clinical stage at diagnosis (0–IV), and facility location. Patient age was organized by decade. Participant race and ethnicity were extracted from patient charts by Certified Tumor Registrars who report data according to state requirements. Race and ethnicity were examined due to previously documented health disparities during the pandemic.^18^, ^19^ Facility location was organized by Census Bureau division.^20^ Stage 0–IV cancers were included in the analysis according to the American Joint Committee on Cancer (AJCC) 8th edition staging system.

### 2.5 Data source

#### 2.5.1 The National Cancer Database (NCDB)

The NCDB is a clinical oncology database. CoC‐accredited centers submit their data to the NCDB, which contains information on the diagnosis and treatment of breast cancer patients and is updated on an annual basis. The NCDB captures approximately 72% of all types of newly diagnosed cancers in the U.S.^21^

#### 2.5.2 Census population data

U.S. Census annual state population estimates for females and males for each year between 2015 and 2020 were collected from the census.gov website. Data were collected on patient age, sex, race, and Hispanic origin and were categorized by Census Bureau division.

### 2.6 Statistical analysis

Demographic characteristics were collected for the NCDB cancer cohort and the Census population cohort. The absolute number of newly diagnosed breast cancer cases was identified and used to estimate the incidence of breast cancer cases from 2015 to 2020 by calculating the number of breast cancer cases per 100,000 individuals based on U.S. Census data. Trends were stratified by year of diagnosis, age, race and ethnicity, and U.S. Census division (state of residence at the time of diagnosis). Data were organized geographically by Census division in order to account for a fluctuating number of CoC‐accredited facilities included in the NCDB over time and to understand the regional impact of COVID‐19 on the U.S. population and breast cancer case numbers. A sensitivity analysis of CoC facilities who reported data to the NCDB for all years during the study period was conducted to determine whether the changing number of facilities included in the NCDB affected the number of breast cancer cases reported each year.

Descriptive statistics are reported as the number of cases with corresponding percentage or mean ± standard deviation (SD). All statistical analysis was performed using SAS 9.4 (SAS Institute, Cary, NC). This study followed the STROBE reporting guideline for cohort studies.

## 3 RESULTS

### 3.1 NCDB and census data demographics

There were 1,487,183 females and 12,623 males diagnosed with breast cancer over the study period. Table 1 demonstrates the demographic and clinical characteristics of breast cancer patients included in the NCDB. Most patients were white (*N* = 1,115,904 females, 75%; *N* = 9709 males, 76.9%) with a mean age of 62 ± 13 years for females and 66 ± 13 years for males. Patients were geographically concentrated in the Middle Atlantic, South Atlantic, and East North Central divisions of the U.S. Most patients had early‐stage breast cancer (*N* = 214,690 females, 83.1%, *N* = 1674 males, 76.2%). Relative to the NCDB dataset, patients captured by the U.S. Census were more likely to be young and non‐white (Table S1).

**TABLE 1:** Characteristics of NCDB Cohort (2015–2020).

Characteristics	NCDB Females	NCDB Males
Total patients	1,487,183	12,623
Age, years [*N* (%)]		
< 30	7629 (0.5)	47 (0.4)
30–39	57,924 (3.9)	293 (2.3)
40–49	214,484 (14.4)	965 (7.6)
50–59	341,878 (23.0)	2237 (17.7)
60–69	429,583 (28.9)	3756 (29.8)
70–79	308,275 (20.7)	3391 (26.9)
≥ 80	127,410 (8.6)	1934 (15.3)
Race/Ethnicity [*N* (%)]		
Non‐Hispanic White	1,115,904 (75.0)	9709 (76.9)
Non‐Hispanic Black or African American	178,741 (12.0)	1824 (14.4)
Hispanic Origin	96,140 (6.5)	521 (4.1)
Non‐Hispanic Asian/Pacific Islander	67,769 (4.6)	356 (2.8)
Non‐Hispanic Other	28,629 (1.9)	213 (1.7)
Insurance [*N* (%)]		
Private	722,688 (48.6)	4715 (37.4)
Medicare	608,330 (40.9)	6604 (52.3)
Medicaid	97,922 (6.6)	633 (5.0)
Other government	16,706 (1.1)	269 (2.1)
Unknown	18,392 (1.2)	183 (1.4)
Uninsured	23,145 (1.6)	219 (1.7)
Facility location [N (%)]		
New England	90,717 (6.1)	933 (7.4)
Middle Atlantic	237,177 (15.9)	2156 (17.1)
South Atlantic	329,132 (22.1)	2723 (21.6)
East North Central	244,942 (16.5)	2247 (17.8)
East South Central	90,424 (6.1)	730 (5.8)
West North Central	106,254 (7.1)	842 (6.7)
West South Central	126,536 (8.5)	1127 (8.9)
Mountain	69,329 (4.7)	542 (4.3)
Pacific	192,672 (13.0)	1323 (10.5)

Abbreviation: NCDB, National Cancer Database.

### 3.2 Breast cancer cases among females in the U.S. from 2015 to 2020

**FIGURE 1:**
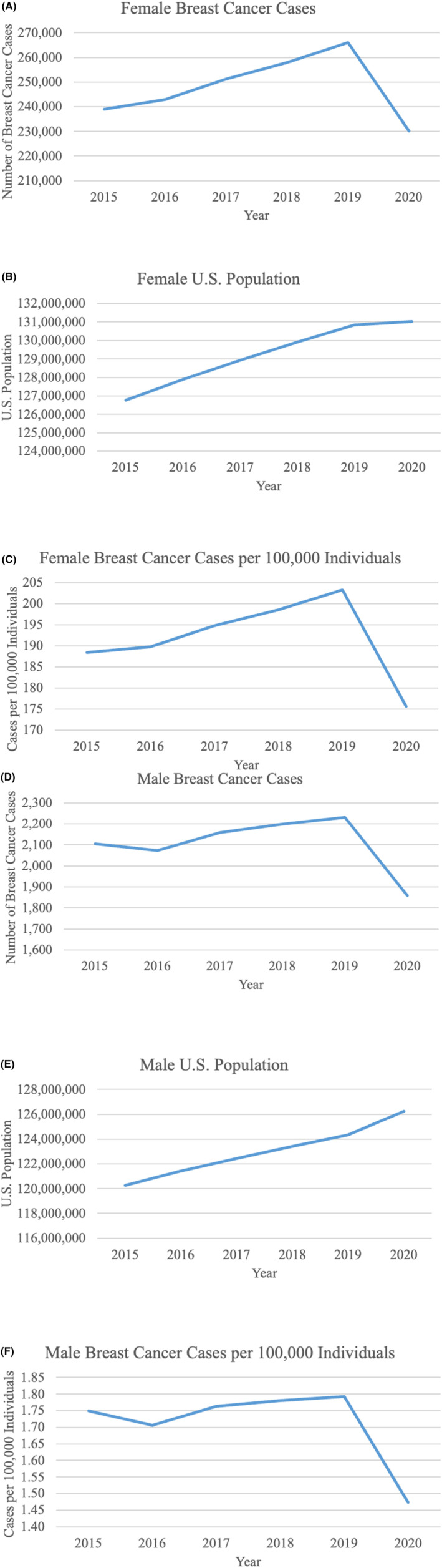
Breast cancer cases in females and males in the U.S. from 2015 to 2020. (A) Breast cancer cases in U.S. females from 2015 to 2020. Cases dropped dramatically from 2019 to 2020. (B). The U.S. female population increased each year between 2015 and 2020. (C) Female breast cancer cases per 100,000 individuals increased each year until 2019. From 2019 to 2020, rates decrease drastically. (D) Breast cancer cases in U.S. Males from 2015 to 2020. As with females, male breast cancer cases dropped between 2019 and 2020. (E) The U.S. male population increased each year between 2015 and 2020. (F) Male breast cancer cases per 100,000 individuals decreased between 2019 and 2020.

Breast cancer cases among females increased from 238,937 in 2015 to 265,980 in 2019 and then decreased to 230,164 cases in 2020 (Figure 1A). There was a 15.6% drop in the absolute number of breast cancer cases from 2019 to 2020 (Table S2). The U.S. female population increased each year from 2015 to 2020 (Figure 1B). Cancer cases per 100,000 females also increased each year from 2015 to 2019 but then declined from 203 in 2019 to 176 in 2020, translating to a 15.7% decrease in cases per 100,000 females from 2019 to 2020 (Figure 1C, Table S2).

**FIGURE 2:**
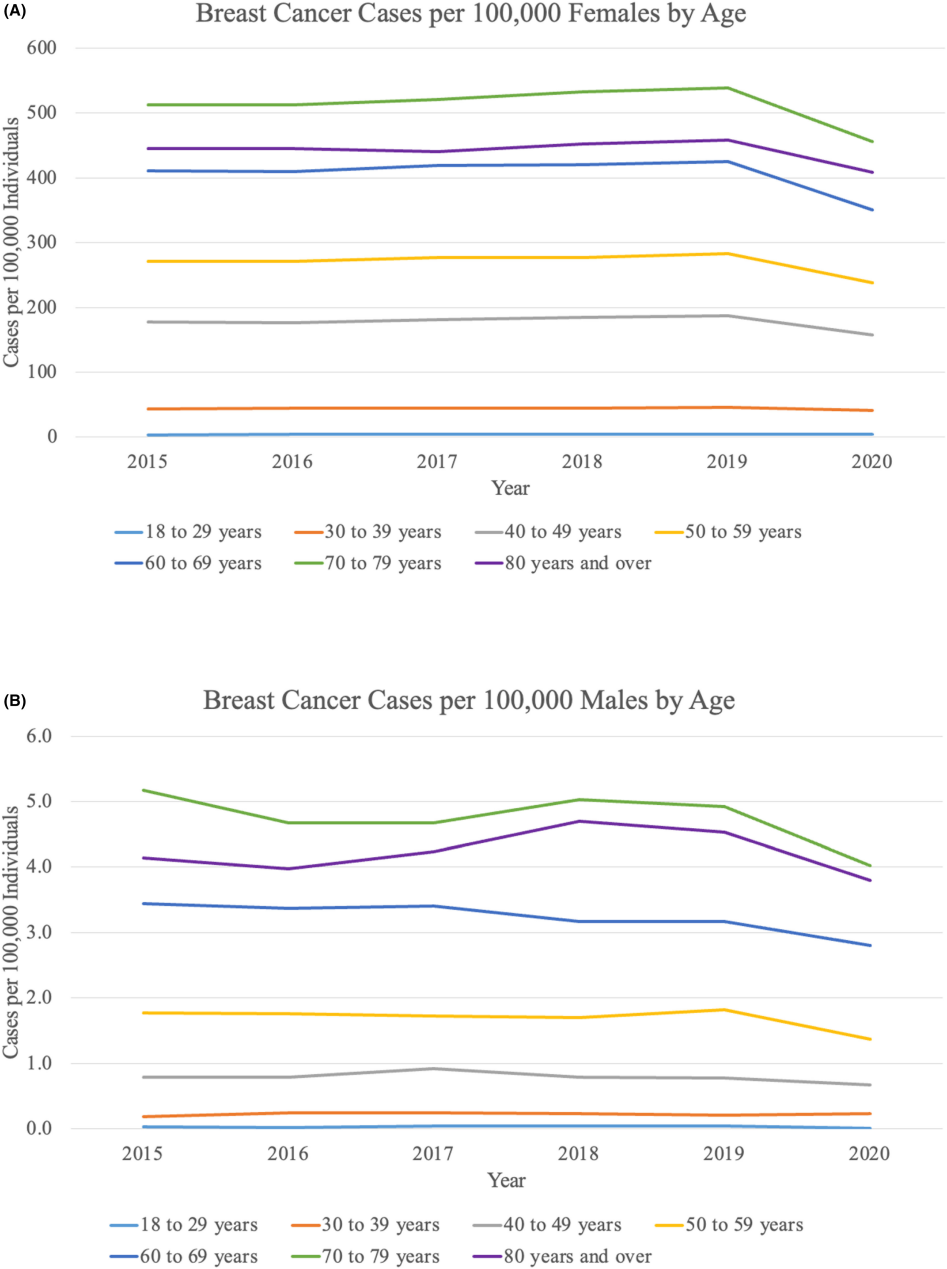
Female and male breast cancer cases by age. (A) Breast cancer cases per 100,000 females stratified by age by decade. (B) Breast cancer cases per 100,000 males stratified by age by decade.

**FIGURE 3:**
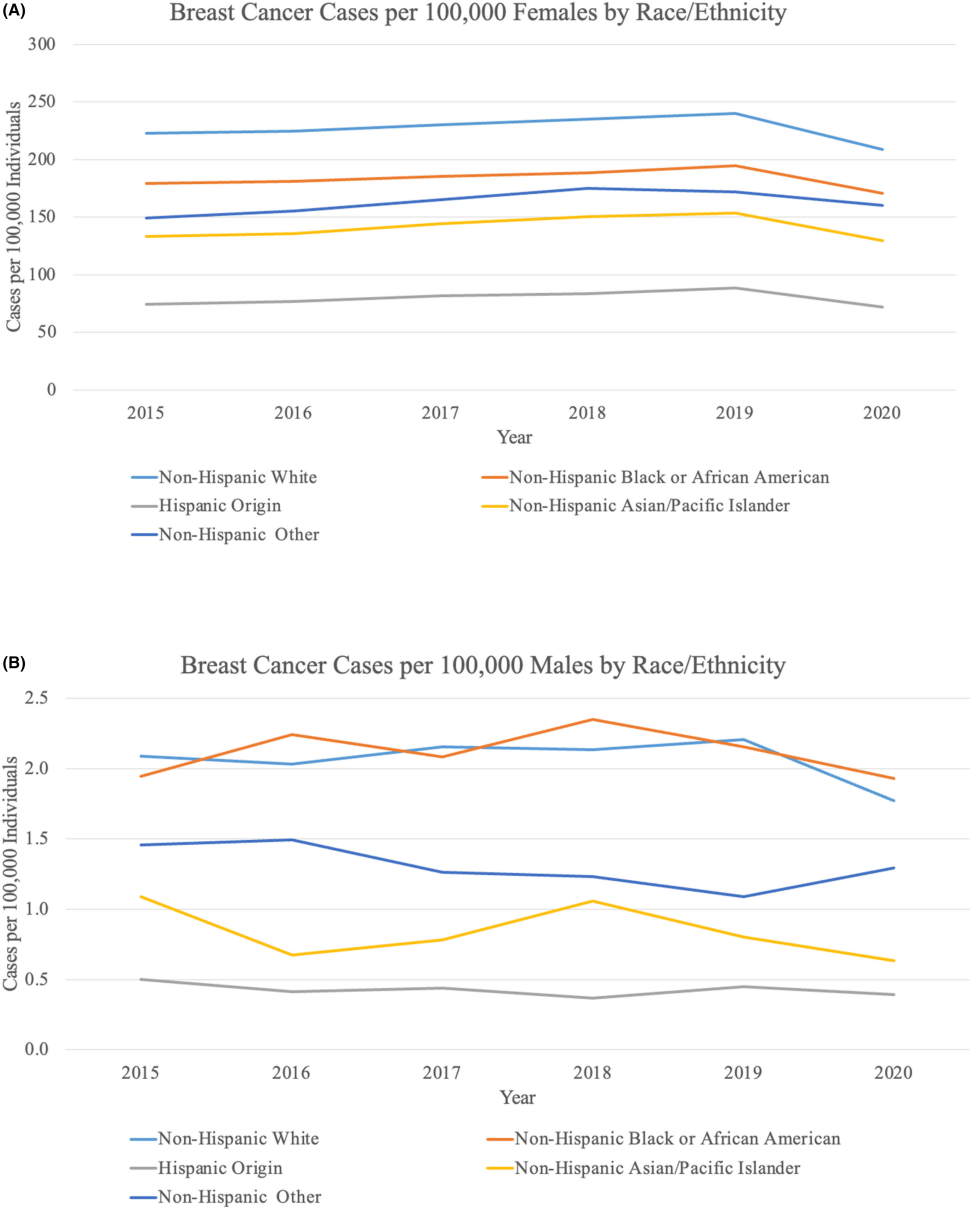
Female and male breast cancer cases by race. (A) Breast cancer cases per 100,000 females stratified by race and ethnicity. (B) Breast cancer cases per 100,000 males stratified by race and ethnicity.

**FIGURE 4:**
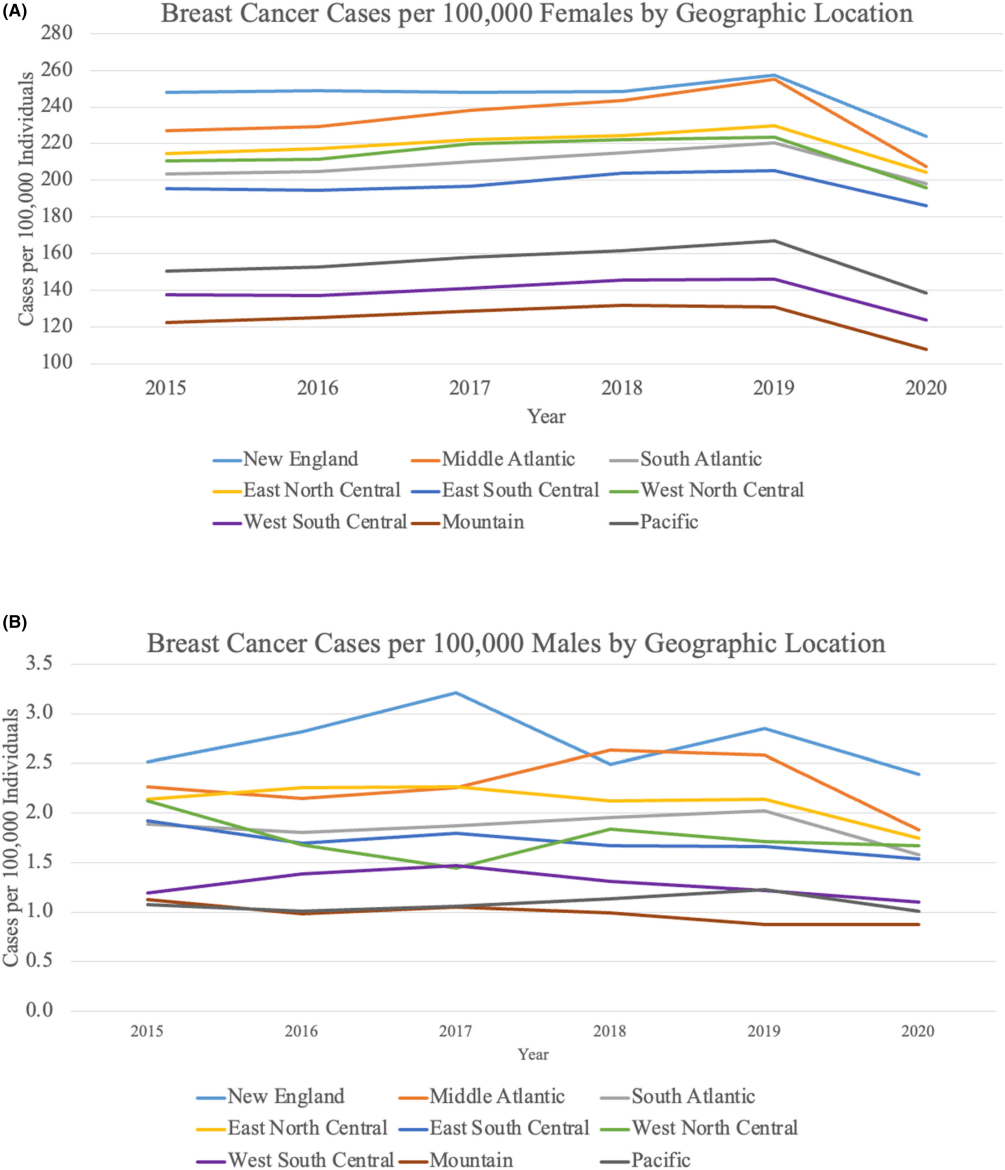
Female and male breast cancer cases by geographic location. (A) Breast cancer cases per 100,000 females stratified by geographic location (U.S. Census Division). (B) Breast cancer cases per 100,000 males stratified by geographic location (U.S. Census Division).

For all age groups except females <40 years old, cases per 100,000 remained stable from 2015 to 2019 and then decreased from 2019 to 2020 (Figure 2A). The decrease was most dramatic for females aged 50–59 years (16.1% decrease) and 60–69 years (17.4% decrease). From 2019 to 2020, cases per 100,000 individuals decreased for all races and all geographic locations (Figures 3A and 4A). The largest decreases were demonstrated in Hispanic patients with an 18.4% decrease from 2019 to 2020 and in patients from the Middle Atlantic division (18.6% decrease).

### 3.3 Breast cancer cases among males in the U.S. from 2015 to 2020

The number of breast cancer cases in males increased from 2015 to 2019 (Figure 1D). Cases then decreased from 2230 in 2019 to 1859 in 2020, equating to a 20% drop (Table S2). The U.S. male population increased each year from 2015 to 2020 (Figure 1E). Cases per 100,000 males increased from 2015 to 2019 but then dropped from 1.8 in 2019 to 1.5 in 2020, translating to a 21.8% decrease in cases per 100,000 males from 2019 to 2020 (Figure 1F, Table S2).

When categorized by age by decade, cases per 100,000 males remained stable or decreased from 2019 to 2020 (Figure 2B). There were clear declining trends in males ≥ 50 years old between 2019 and 2020, and the largest difference was seen among males aged 50–59 years with a 25.0% decrease. There was no significant trend in incidence by race or geographic location from 2015 to 2020 (Figures 3B and 4B). Nonetheless, cases per 100,000 males decreased from 2019 to 2020 for patients of all races except for “Non‐Hispanic Other” and for all geographic locations except for the Mountain and West North Central divisions, which remained stable.

### 3.4 Breast cancer stage distribution from 2018 to 2020

**FIGURE 5:**
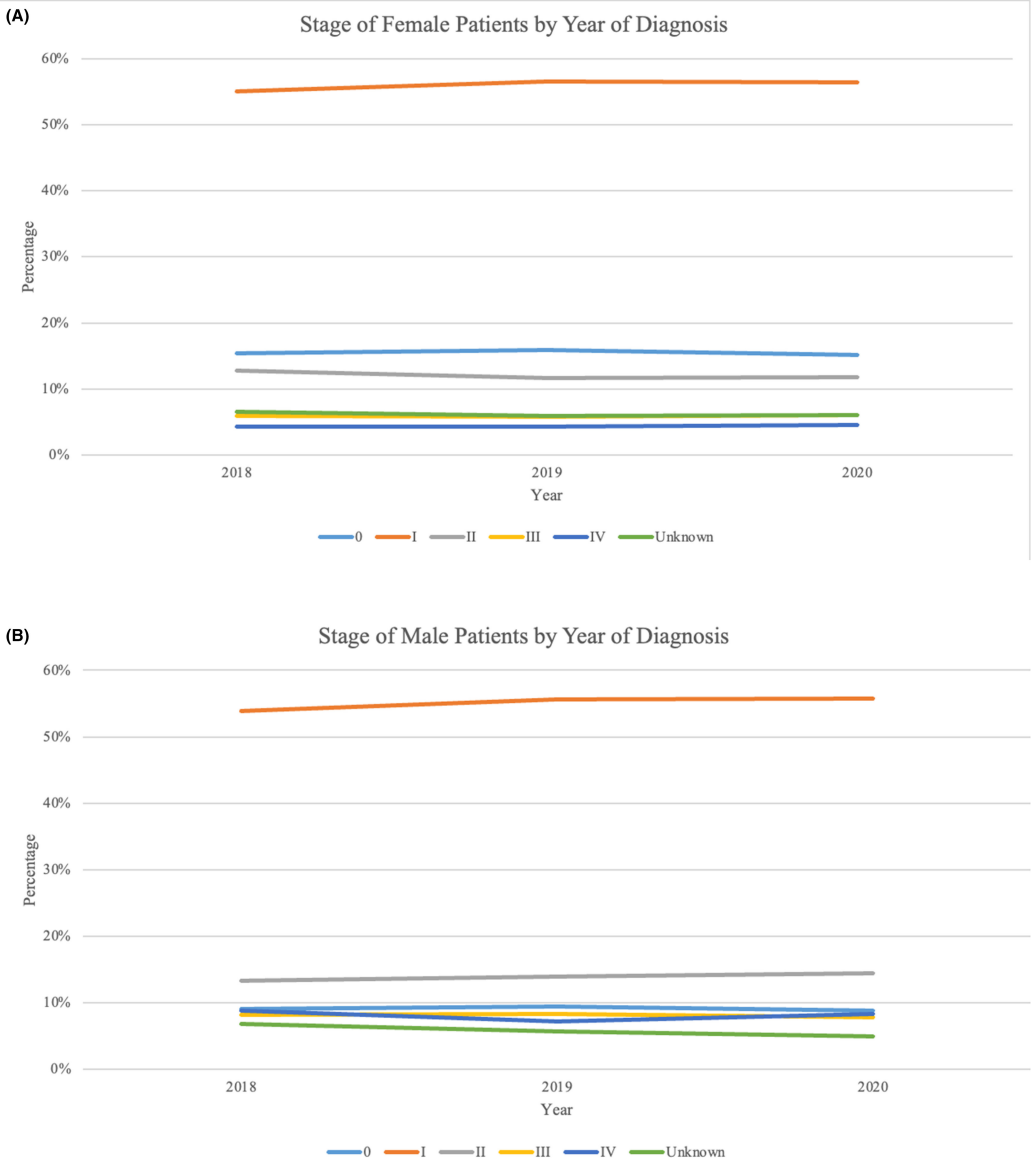
Breast cancer stage at the time of diagnosis (2018–2020). (A) Cancer stage at the time of diagnosis for female patients. (B) Cancer stage at the time of diagnosis for male patients.

The stage at presentation was examined from 2018 to 2020. This analysis was confined to these years because of changes in AJCC staging that occurred in 2018, making it difficult to compare to earlier years. Cancer stage distribution remained stable from 2018 to 2020 for both females and males (Figure 5). When female patients were stratified by age, race and ethnicity, and geographic location, stage distribution did not meaningfully vary from year to year.

### 3.5 Sensitivity analysis of incidence of breast cancer at CoC sites with 5 years of data

In 2015, there were 1287 CoC‐accredited facilities that reported a total of 241,041 newly diagnosed breast cancer cases. This increased to 1306 facilities in 2019 reporting 268,210 cases and then decreased to 1280 facilities in 2020 reporting 232,023 cases. There were 1242 facilities out of a total of 1328 facilities included in the NCDB that reported cases every year from 2015 to 2020. Using data from these facilities alone, the percent change from 2019 to 2020 for incidence was slightly smaller but illustrated a similar trend to the percent change when examining all 1338 facilities (12.5% decrease for females at facilities that reported data every year versus 15.7% decrease for females at all CoC facilities; 17.3% decrease for males at facilities that reported data every year versus 21.8% decrease for males at all CoC facilities) (Table S3).

## 4 DISCUSSION

This is one of the first large‐scale studies to examine the rates of breast cancer diagnosis during the first year of the COVID‐19 pandemic in the U.S. Our study demonstrates an approximate 15–20% decrease in newly diagnosed breast cancer cases reported to CoC sites in 2020 compared to 2019. The decrease was seen for both females and males and across nearly all patient age groups, races and ethnicities, and Census divisions.

Only a few studies have examined the number of breast cancer cases diagnosed during the pandemic.^6^, ^22^, ^23^, ^24^, ^25^ Most of these studies used databases that did not account for a majority of cancer cases or were single institution studies. A recently published study examining all cancer types used the NCDB to reveal a 13% decrease in new breast cancer cases during the pandemic.^6^ Other studies have demonstrated a decrease in breast cancer incidence by nearly 50% during the early part of the pandemic compared to baseline.^22^, ^25^

Our study relies on the data reported by CoC facilities, which captures approximately 72% of all newly diagnosed cancer cases every year in the U.S.^21^ Census data was used as the denominator to calculate breast cancer incidence in order to adjust for geographical population change. Additionally, these two databases were selected because the American Cancer Society only provides model‐based projections of breast cancer incidence based on data from prior years, making it suboptimal for this study's objective.

From 2015 to 2020, the number of facilities reporting data to the NCDB changed each year by approximately −0.5%. When CoC sites that reported data consistently over all 5 years of the study were examined, there was a similar incidence drop between 2019 and 2020 compared to the drop when all CoC sites were included (Tables S2 and S3). This finding suggests that the demonstrated change in incidence from 2019 to 2020 was not related to small yearly changes in the number of CoC facilities included in the NCDB. Additionally, a recent study showed that cancer cases were reliably reported to the NCDB during the pandemic and thus the incidence decline was not secondary to an inability to extract data during a state of emergency.^26^

The reasons for a decrease in new breast cancer cases during the pandemic are multifactorial. First, there were explicit government and societal guidelines that recommended avoiding the health care system when possible or delaying the diagnosis and treatment of breast cancer, particularly in the acute phases of the pandemic. In March 2020, the American Society of Breast Surgeons and the American College of Radiology recommended that centers “postpone all breast screening exams effective immediately”.^27^ Meanwhile, 42 states and territories mandated stay‐at‐home orders between March and May of 2020.^28^ Because of these policies, many patients did not get their screening mammogram like they otherwise would have. In addition, patients were avoiding medical care to abide by the stay‐at‐home orders and not being evaluated even if they had a self‐detected breast mass. Beyond these guidelines, patients experienced fear and anxiety associated with COVID‐19 exposure, which deterred many from seeking health care even after the stay‐at‐home orders lifted. A Centers for Disease Control and Prevention report estimated that 41% of U.S. adults delayed or avoided urgent/emergent and routine health care by June 2020 with a disproportionate number of Black and Hispanic adults avoiding care.^29^ Even as COVID‐19 numbers decrease and restrictions fade, fear and factors associated with the pandemic such as unemployment may cause patients to be persistently reluctant or unable to seek health care.

It is clear that the pandemic created large gaps in breast cancer screening and diagnostic procedures.^7^, ^30^, ^31^ In an analysis of more than five million screening mammograms, only 36% of pre‐COVID mammogram volumes were performed during peak COVID times.^22^ It is estimated that approximately 40–60% of all newly diagnosed breast cancer is detected by screening mammogram.^32^, ^33^ With such a large proportion of breast cancer detected via screening, the screening deficit almost certainly led to a reduction in diagnostic mammograms and biopsies, partially accounting for the decrease in diagnoses. This study demonstrates an approximate 20% decrease in breast cancer cases early in the pandemic which is far below the percentage of breast cancer cases diagnosed through screening each year. However, these data are cumulative over the year of 2020, and the screening deficit may have varied on a monthly basis and gradually improved throughout the year as the burden of COVID‐19 declined. The full effect of the screening deficit in 2020 has yet to be appreciated and longer follow up is required to understand the true extent of the pandemic's impact. Furthermore, it is not clear whether the screening deficit is due to patients not coming in to receive health care or if patients had adequate interaction with health care providers but were faced with limited availability for screening tests related to the pandemic. Unfortunately, the NCDB does not contain any screening information, so we cannot examine how the pandemic impacted screening for breast cancer.

In this study, both females and males demonstrated declines from 2019 to 2020, however, the smaller sample size for males made it difficult to show the clear trends that were demonstrated in females. Nonetheless, the analysis in male patients showed similar declines. Because males do not undergo screening for breast cancer, these data suggest that the incidence decrease is secondary to males not coming in to be evaluated by their provider. Female patients aged 60‐69 years experienced the largest decrease from 2019 to 2020 out of all the age groups examined. At age 40–50, average‐risk women begin screening for breast cancer, which may account for this finding. Additionally, patients over the age of 50 are more likely to be affected by breast cancer compared to younger age groups. This trend may also reflect a disproportionate decrease in health care utilization among older adults during the pandemic. The year 2020 marked the first time in over 20 years that Medicare spending declined, emphasizing the large impact that the pandemic had on older adults.^34^

Hispanic females had the sharpest decline from 2019 to 2020 among all races and ethnicities, with an 18.4% decrease. This is consistent with other disparities that Hispanic individuals faced during the pandemic—Hispanics had higher rates of COVID‐19 infection as well as mortality related to COVID‐19 compared to non‐Hispanic Whites.^18^, ^19^, ^29^, ^35^, ^36^

Female patients receiving care in the western U.S. and the Middle Atlantic division experienced the largest declines in 2020, which may reflect the large COVID‐19 outbreaks in New York, Washington State, and California at the time of the study period. One study examined the screening deficit for three different types of cancers using a cohort of 60 million people and also found that the Northeast region experienced the largest declines in screening.^7^

This analysis showed that cancer stage did not vary between 2018 and 2020. When stage was stratified by age, race and ethnicity, and geographic location, there were no differences for any subgroup between years. This study is limited in that 2020 is the most recent year available in the NCDB, and one year may not be enough time to note a stage difference in the U.S. The decrease in cancer cases during the pandemic may equate to a delay in diagnosis and eventually lead to patients presenting at more advanced stages.^23^, ^37^, ^38^

The strengths of this study include the large volume of patients from across the country and the ability to examine trends across subgroups such as different ages and races. Limitations include the fact that this study only examined cases reported to CoC sites, which may not be representative of the typical facility treating patients with breast cancer in the U.S. and may include sites with larger volumes of patients and more resources. The NCDB does not include monthly case numbers, which makes it impossible to understand the duration of the drop in diagnoses in 2020. It is also a retrospective database and lacks detailed patient information. Finally, stage distribution could only be evaluated from 2018 to 2020 because the AJCC staging system changed in 2018.

## 5 CONCLUSION

These findings demonstrate decreased rates of breast cancer diagnosis in the U.S. during the first year of the COVID‐19 pandemic. The decreases are sustained for female and male patients and for almost all age groups, races and ethnicities, and geographic locations. Hispanic patients and patients receiving care at CoC facilities in the Middle Atlantic division of the U.S. experienced the largest decline between 2019 and 2020. Identifying the impact of the pandemic on breast cancer patients allows physicians and policymakers to recognize and address issues that may arise secondary to diagnostic delays. Future research is needed to determine when case numbers will return to baseline levels and what the consequences will be of delayed diagnosis and care, particularly among patients and geographic areas that were most severely affected by the pandemic.

## AUTHOR CONTRIBUTIONS

**Marie Fefferman:** Conceptualization (equal); data curation (supporting); formal analysis (supporting); investigation (equal); methodology (equal); writing – original draft (lead); writing – review and editing (equal). **Kristine Kuchta:** Data curation (equal); formal analysis (equal); methodology (equal); writing – review and editing (supporting). **Chi‐Hsiung Wang:** Formal analysis (equal); methodology (equal); writing – review and editing (supporting). **Kyra Nicholson:** Conceptualization (supporting); investigation (supporting); writing – review and editing (supporting). **Katherine Kopkash:** Conceptualization (supporting); supervision (supporting); writing – review and editing (supporting). **Catherine Pesce:** Conceptualization (supporting); supervision (supporting); writing – review and editing (supporting). **Elizabeth Poli:** Conceptualization (supporting); supervision (supporting); writing – review and editing (supporting). **Thomas W. Smith:** Conceptualization (supporting); supervision (supporting); writing – review and editing (supporting). **Katharine Yao:** Conceptualization (equal); data curation (equal); investigation (equal); methodology (equal); project administration (lead); resources (lead); supervision (lead); writing – original draft (supporting); writing – review and editing (equal).

## FUNDING INFORMATION

No external funding was used for this study.

## CONFLICT OF INTEREST STATEMENT

None.

## ETHICS STATEMENT

Ethics approval was not required or obtained for this study. Informed consent was not obtained.

## CONSENT

Patient consent was not obtained given the use of de‐identified data through the National Cancer Database.

## Supporting information


Table S1.

## Data Availability

The authors have a data use agreement with the American College of Surgeons that allows for the use of NCDB data. The U.S. population data used for this study is publicly available and may be accessed from the U.S. Census Bureau at www.census.gov.
