# Supplementary material for: Rates of newly diagnosed breast cancer at commission on cancer facilities during the early phase of the COVID‐19 pandemic
Source: Cancer Med. 2023 Dec 22;13(1):e6874. doi: 10.1002/cam4.6874 (PMC10807625; doi:10.1002/cam4.6874)
Supplement: Supplementary file 1 — Table S1. [file CAM4-13-e6874-s001.docx]

**SUPPLEMENTARY TABLES & FIGURES**

**Supplementary Table 1. Comparison of Characteristics of Patients included in the NCDB (2015-2020) vs. the aggregate U.S. population (2015-2020), Age ≥ 18**

|  | **NCDB Females** | **U.S. Census Females** | **NCDB Males** | **U.S. Census Males** |
| --- | --- | --- | --- | --- |
| **Total Patients** | 1,487,183 | 738,143,976 | 12,623 | 775,413,403 |
| **Age, years [N (%)]** |  |  |  |  |
| < 30 | 7,629 (0.5) | 164,256,826 (22.3%) | 47 (0.4) | 157,342,962 (20.3%) |
| 30-39 | 57,924 (3.9) | 131,026,123 (17.8%) | 293 (2.3) | 129,531,403 (16.7%) |
| 40-49 | 214,484 (14.4) | 121,024,590 (16.4%) | 965 (7.6) | 122,644,748 (15.8%) |
| 50-59 | 341,878 (23.0) | 126,892,742 (17.2%) | 2,237 (17.7) | 132,058,881 (17.0%) |
| 60-69 | 429,583 (28.9) | 105,914,898 (14.3%) | 3,756 (29.8) | 116,545,267 (15.0%) |
| 70-79 | 308,275 (20.7) | 60,199,820 (8.2%) | 3,391 (26.9) | 71,622,474 (9.2%) |
| ≥ 80 | 127,410 (8.6) | 28,828,977 (3.9%) | 1,934 (15.3) | 45,667,668 (5.9%) |
| **Race/Ethnicity [N (%)]** |  |  |  |  |
| Non-Hispanic White | 1,115,904 (75.0) | 470,736,046 (63.8%) | 9,709 (76.9) | 491,620,220 (63.4%) |
| Non-Hispanic Black or African American | 178,741 (12.0) | 86,204,878 (11.7%) | 1,824 (14.4) | 97,5464,59 (12.6%) |
| Hispanic Origin | 96,140 (6.5) | 122,315,337 (16.6%) | 521 (4.1) | 120,725,476 (15.6%) |
| Non-Hispanic Asian/Pacific Islander | 67,769 (4.6) | 42,518,734 (5.8%) | 356 (2.8) | 47,977,033 (6.2%) |
| Non-Hispanic Other | 28,629 (1.9) | 16,368,981 (2.2%) | 213 (1.7) | 17,544,215 (2.3%) |
| **Facility Location [N (%)]** |  |  |  |  |
| New England | 90,717 (6.1) | 34,409,478 (4.7%) | 933 (7.4) | 36,918,142 (4.8%) |
| Middle Atlantic | 237,177 (15.9) | 94,422,679 (12.8%) | 2,156 (17.1) | 101,585,543 (13.1%) |
| South Atlantic | 329,132 (22.1) | 146,905,200 (19.9%) | 2,723 (21.6) | 157,735,540 (20.3%) |
| East North Central | 244,942 (16.5) | 106,412,422 (14.4%) | 2,247 (17.8) | 111,947,327 (14.4%) |
| East South Central | 90,424 (6.1) | 42,596,337 (5.8%) | 730 (5.8) | 45,901,658 (5.9%) |
| West North Central | 106,254 (7.1) | 48,261,230 (6.5%) | 842 (6.7) | 49,662,843 (6.4%) |
| West South Central | 126,536 (8.5) | 88,006,356 (11.9%) | 1,127 (8.9) | 91,353,053 (11.8%) |
| Mountain | 69,329 (4.7) | 55,277,448 (7.5%) | 542 (4.3) | 55,749,052 (7.2%) |
| Pacific | 192,672 (13.0) | 121,852,826 (16.5%) | 1,323 (10.5) | 124,560,245 (16.1%) |

Abbreviation: NCDB = National Cancer Database

**Supplementary Table 2. Breast Cancer Cases from 2015 to 2020 from All CoC Facilities**

|  |  | **2015** | **2016** | **2017** | **2018** | **2019** | **2020** | **Percent Change from 2019-2020** |
| --- | --- | --- | --- | --- | --- | --- | --- | --- |
| **Female** | **U.S. Population** | 126,764,800 | 127,888,484 | 128,939,978 | 129,931,126 | 130,851,717 | 131,037,298 | 0.1% |
|  | **Cancer Cases** | 238,937 | 242,793 | 251,248 | 258,061 | 265,980 | 230,164 | -15.6% |
|  | **Cases/100,000 Population** | 188 | 190 | 195 | 199 | 203 | 176 | -15.7% |
| **Male** | **U.S. Population** | 120,252,312 | 121,403,414 | 122,460,215 | 123,437,230 | 124,348,656 | 126,242,149 | 1.5% |
|  | **Cancer Cases** | 2,104 | 2,072 | 2,160 | 2,198 | 2,230 | 1,859 | -20.0% |
|  | **Cases/100,000 Population** | 1.7 | 1.7 | 1.8 | 1.8 | 1.8 | 1.5 | -21.8% |

Abbreviation: CoC= Commission on Cancer

**Supplementary Table 3. Sensitivity Analysis of Breast Cancer Cases from 2015 to 2020 from CoC Facilities that Reported Data to the NCDB Every Year**

|  |  | **2015** | **2016** | **2017** | **2018** | **2019** | **2020** | **Percent Change from 2019 to 2020** |
| --- | --- | --- | --- | --- | --- | --- | --- | --- |
| **Female** | **U.S. Population** | 126,764,800 | 127,888,484 | 128,939,978 | 129,931,126 | 130,851,717 | 131,037,298 | 0.1% |
|  | **Breast Cancer Cases** | 235,109 | 236,533 | 243,761 | 250,213 | 258,694 | 226,788 | -12.3% |
|  | **Cases/100,000 Individuals** | 185 | 185 | 189 | 193 | 198 | 173 | -12.5% |
| **Male** | **U.S. Population** | 120,252,312 | 121,403,414 | 122,460,215 | 123,437,230 | 124,348,656 | 126,242,149 | 1.5% |
|  | **Breast Cancer Cases** | 2,067 | 2,014 | 2,085 | 2,132 | 2,178 | 1,829 | -16.0% |
|  | **Cases/100,000 Individuals** | 1.7 | 1.7 | 1.7 | 1.7 | 1.8 | 1.4 | -17.3% |

Abbreviation: NCDB = National Cancer Database
